# Methylene blue dosing strategies in critically ill adults with shock—A retrospective cohort study

**DOI:** 10.3389/fmed.2022.1014276

**Published:** 2022-10-28

**Authors:** Sibel Sari-Yavuz, Ka-Lin Heck-Swain, Marius Keller, Harry Magunia, You-Shan Feng, Helene A. Haeberle, Petra Wied, Christian Schlensak, Peter Rosenberger, Michael Koeppen

**Affiliations:** ^1^Department of Anesthesiology and Intensive Care Medicine, University Hospital Tübingen, Tübingen, Germany; ^2^Institute for Clinical Epidemiology and Applied Biostatistics (IKEaB), Eberhard-Karls-University Tübingen, Tübingen, Germany; ^3^Department of Thoracic and Cardiovascular Surgery, University Hospital Tübingen, Tübingen, Germany

**Keywords:** shock, methylene blue, critical care, retrospective study, clinical trial

## Abstract

**Background:**

Shock increases mortality in the critically ill and the mainstay of therapy is the administration of vasopressor agents to achieve hemodynamic targets. In the past, studies have found that the NO-pathway antagonist methylene blue improves hemodynamics. However, the optimal dosing strategy remains elusive. Therefore, we investigated the hemodynamic and ICU outcome parameters of three different dosing strategies for methylene blue.

**Methods:**

We performed a retrospective cohort study of patients in shock treated with methylene blue. Shock was defined as norepinephrine dose >0.1 μg/kg/min and serum lactate level >2 mmol/l at the start of methylene blue administration. Different demographic variables, ICU treatment, and outcome parameters were evaluated. To compare the differences in the administration of vasopressors or inotropes, the vasoactive inotropic score (VIS) was calculated at different time points after starting the administration of methylene blue. Response to methylene blue or mortality at 28 days were assessed.

**Results:**

262 patients from July 2014 to October 2019 received methylene blue. 209 patients met the inclusion criteria. Three different dosing strategies were identified: bolus injection followed by continuous infusion (*n* = 111), bolus injection only (no continuous infusion; *n* = 59) or continuous infusion only (no bolus prior; *n* = 39). The groups did not differ in demographics, ICU scoring system, or comorbidities. In all groups, VIS decreased over time, indicating improved hemodynamics. Cardiogenic shock and higher doses of norepinephrine increased the chance of responding to methylene blue, while bolus only decreased the chance of responding to methylene blue treatment. 28-day mortality increased with higher SAPSII scores and higher serum lactate levels, while bolus injection followed by continuous infusion decreased 28-day mortality. No severe side effects were noted.

**Conclusion:**

In this cohort, methylene blue as a bolus injection followed by continuous infusion was associated with a reduced 28-day mortality in patients with shock. Prospective studies are needed to systematically evaluate the role of methylene blue in the treatment of shock.

## Background

Shock results from a multitude of pathophysiological processes and leads to hemodynamic collapse and death if left untreated. Clinicians differentiate forms of shock such as cardiogenic, hypovolemic, obstructive, or distribution shock (including septic shock) (1). They all share the same features, leading to hypotension and impaired oxygen delivery or utilization in the peripheral circulation (2), either due to loss of vascular tone or decreased cardiac output. This jumpstarts a vicious circle consisting of an altered microcirculation leading to the production and release of pro-inflammatory mediators such as tumor necrosis factor alpha (TNFa) or interleukin-6 (IL-6), which in turn induce the expression of the inducible NO-synthase (iNOS) with subsequent production of the vasodilator nitric oxide (NO). NO acts as a vasodilator that directly counteracts the endogenous or exogenous vasoconstrictors and thus decreases the vascular tone (3). Interestingly, increased endothelial NO production contributes substantially to the pathophysiology of shock (4, 5) and leads to a decrease in mean arterial pressure resulting in impaired organ perfusion. If volume resuscitation alone cannot reestablish target values, patients require vasopressors. In recent decades, different vasopressors, such as dopamine, epinephrine, or norepinephrine, have been clinically tested in shock patients (6–8). Norepinephrine has emerged as the first-line vasopressor agent in most forms of shock. In patients with severe circulatory failure, additional vasoactive substances are required to stabilize the blood pressure. Several clinical studies have evaluated NO-synthase inhibitors in experimental and clinical shock treatment (9), but the use of these substances never took hold in clinical therapy.

In 1992, patients with septic shock (a form of distributive shock) received the first fully synthetic drug ever used in medicine invented in 1876 (10): methylene blue. Methylene blue was clinically tested as a shock treatment in various clinical trials (11, 12) evaluating different dosing strategies for methylene blue dosing regimens. They range from bolus administration regimens to bolus administration followed by continuous infusion, or continuous infusion only without additional bolus (13). To date, no studies have investigated the optimal dose strategy for methylene blue in critically ill patients with shock, mainly due to the complex pharmacological properties of methylene blue. It has a large volume of distribution (up to 250 l) and a long half-life of up to 24 h (13), making the prediction of clinical effects and hemodynamic responses difficult.

At our institution, the administration of methylene blue belongs to the standard operating procedure in the treatment of refractory shock. Three different dosing strategies have been used: bolus of methylene blue followed by continuous infusion, bolus injection only, or continuous infusion only. In the present retrospective cohort analysis, we performed an unbiased analysis of these three different regimens and investigated whether one dosing strategy leads to improved hemodynamic stability over the other. Next, we searched for variables that correlate with a clinical response to methylene blue, defined as a reduction in vasoactive or inotropic substances. Finally, we assessed which clinical variables correlate with reduced mortality at 28 days in critically ill adults.

## Methods

### Study population and ethics

All data were retrospectively collected from medical records from the University Hospital of Tuebingen. The study was approved by the Ethics Committee of the University Hospital Tuebingen (IRB# 768/2018BO2), which waived the need for informed consent because patient anonymity was maintained. All methods were approved by the local IRB and performed in accordance with the Declaration of Helsinki and the relevant guidelines.

### Methylene blue therapy

In our institution, patients received methylene blue therapy according to the standard operating procedure (SOP). At the discretion of the attending physician, methylene blue *can be considered* if norepinephrine dose increases >0.1 μg/kg/min. When norepinephrine administration does not establish sufficient hemodynamics (norepinephrine >0.3 μg/kg/min), vasopressin at a dose of 0.06 IU/kg/min is added for vasopressor therapy. If mean arterial pressure cannot be maintained at more than 65 mmHg, methylene blue is added to the therapy by standard.

Three different dosing strategies have been used in our institution in the past: bolus injection alone, continuous infusion without bolus, injection, or bolus injection followed by continuous infusion (bolus 2 mg/kg; continuous infusion with 0.25 mg/kg/h). We define a patient as a “methylene blue responder” according to the following criteria: VIS decreases by >10% within 3 h after administration and mean arterial pressure >65 mmHg.

### Data collection

All patient records who were treated at the Department of Anesthesiology and Intensive Care Medicine between July 2014 and October 2019 were retrospectively screened for inclusion in this observational cohort study. We selected all patients who received methylene blue during their stay in the ICU. We included patients with the following inclusion criteria: Norepinephrine dose 0.1 μg/kg/min, serum lactate 2 mmol/l and methylene blue treatment. The following exclusion criteria were applied: Age < 18 years, no vasopressor or inotropic therapy, administration of methylene for other reasons than hemodynamic compromise.

Based on data from the clinical information system, a database was generated that contains relevant patient information, including age, sex, date of admission and discharge from the ICU. Furthermore, we recorded ICU prognosis scores, such as Sequential Organ Failure Assessment (SOFA), Simplified Acute Physiology Score II (SAPS II) and Acute Physiology and Chronic Health Evaluation (APACHE) scores. All scores presented were calculated for the day of methylene blue administration. We determined comorbidities from the patient records as well as the dosage of vasopressor or inotropic therapy.

All ICU progress and discharge notes were screened for severe side effect of methylene blue. The most severe form of side effects is anaphylaxis, followed by shortness of breath, nausea, vomiting. The most common side effect is a bluish to greenish discoloration of the skin or urine, which is self-resolving (14).

### Classification of shock

Based on the discharge note from the ICU, we retrospectively classified the patients into a category based on available parameters. Patients with documented infection and shock were classified as septic shock. Patients with extended hemodynamic monitoring and a cardiac index < 2.5 l/min/m^2^ were classified as cardiogenic shock. Patients with a shock criterion as described above, but could not be categorized into one of the other two categories, were classified as vasoplegic syndrome.

### Vasoactive inotropic score

In the present study, different vasoactive substances were used. Often patients received different substances at once. These can be subdivided into two broad categories, inotropes and vasopressor. As inotropes, dobutamine and milrinone were used. In the category of the vasopressors, norepinephrine is considered the first-line treatment option; vasopressin is considered additionally. Epinephrine is used in the clinical setting for both indications. Most patients received more than one substance of the respective category. Furthermore, vasoactive and inotropic doses can differ greatly between institutions. To quantify the degree of hemodynamic support objectively, we calculated the so-called *vasoactive inotropic score* (VIS) as described (15). The VIS incorporates vasoconstrictor and inoptropes in a single variable. The VIS was calculated using the following formula:


(1) $$\begin{array}{l} VIS=Dopamine\left(\text{μ}g/kg/min\right)+Dobutamine\left(\text{μ}g/kg/min\right)\\ \;+\left[100\times Epinephrine\left(\text{μ}g/kg/min\right)\right]\\ \;+\left[10.000\times Vasopressin\left(U/kg/min\right)\right]\\ \;+\left[100\times Norepinephrine\left(\text{μ}g/kg/min\right)\right]\\ \;+\left[10\times Milrinon\;\left(\text{μ}g/kg/min\right)\right]. \end{array}$$

### Statistical analysis

Data for continuous variables are expressed as the mean ± standard deviation in the case of a normal distribution and as the median (interquartile range) in the case of a nonnormal distribution. Variables from the different groups were compared using the ANOVA test with Tukey-Kramer *post-hoc* test for multiple comparison. Categorical variables expressed as proportions were compared using the chi-square test. To assess the association of different variables with the response status of methylene blue or mortality at 28 days, univariate logistic regression was used. Variables with biologic plausibility to influence the response status to methylene blue or mortality with a significant association in univariate regression (defined as *p* < 0.1) were considered for inclusion in a multivariate logistic regression model. Collinearity was tested using variance inflation factors and condition indices. If two or more variables showed collinearity, the variable with the lowest *p*-value in the univariate logistic regression was included in the multivariate model. Residuals were tested for normal distribution. Goodness-of-fit was tested using the Hosmer-Lemeshow test. In the logistic regression analysis, we performed full-data set analysis. No missing data was noted for the variables integrated in the univariate or multivariate logistic regression. All statistical analyzes for this study were performed in Prism 9 (GraphPad Software Inc.) and JMP 16.0 (SAS Institute Inc., Cary, US). The *p* values are two-tailed and values < 0.05 are considered statistically significant.

## Results

### Patient selection and characteristics

**Figure 1:**
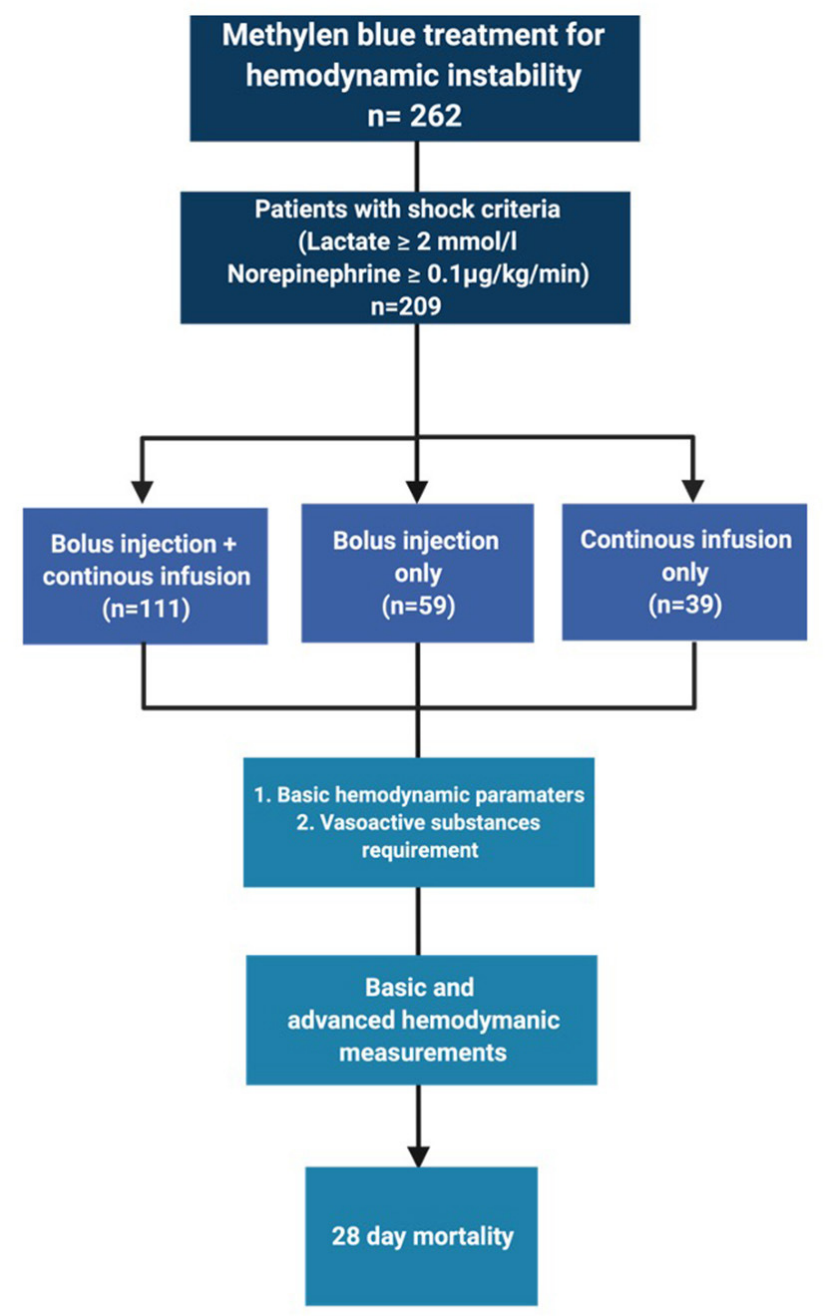
Patient selection strategy.

The selection process is depicted in Figure 1. In total, we identified 209 patients for analysis.

### Demographic data

In our dataset, we identified three different dosing strategies: 53% (*n* = 111) of the patients received a bolus injection followed by continuous infusion. 28% of the patients received only a bolus injection (*n* = 59) and 19% received only a continuous infusion of methylene blue without prior bolus (*n* = 39). Baseline demographic characteristics, such as age, sex, height, weight, and body mass index, were similar between these groups (Table 1). The distribution across types of shock did not differ between the three different dosing strategies.

**Table 1:** Demographic data and comorbidities in shock patients.

	**Total (*n* = 209)**	**Bolus + infusion (*n* = 111)**	**Bolus only (*n* = 59)**	**Continuous infusion only (*n* = 39)**	***p*-values**
Percentage of total		53	28	19	
Age–year (mean ± SD)	65.0 ± 13.7	64.5 ± 13.9	67.9 ± 12.6	62.5 ± 14.4	0.1279
Male sex–no. (%)	74.1%	75.7%	72.9%	71.8%	0.8623
Height–cm (mean ± SD)	173.2 ± 9.5	174.3 ± 9.2	172.8 ± 10.0	170.9 ± 9.3	0.1566
Weight–kg (mean ± SD)	84 ± 19	86 ± 21	85 ± 18	79.4 ± 11.8	0.1962
Body Mass Index (kg/m^2^)	28.1 ± 5.7	28.2 ± 6.4	28.4 ± 5.2	27.1 ± 3.5	0.5232
**Etiology of shock** ***n*** (% in group)					
Sepsis	82 (39.2)	40 (36.0)	24 (40.7)	18 (46.1)	0.5192
Cardiogenic	64 (30.6)	31 (27.9)	20 (33.9)	13 (33.3)	0.6662
Vasoplegia	63 (30.1)	40 (36.0)	15 (25.4)	8 (20.5)	0.1242
Comorbidities					
**Lung disease (%)**	29 (13.8)	11 (09.0)	13 (24.5)	5 (14.3)	0.0153
Coronary artery disease	171 (81.8)	104 (85.9)	41 (77.3)	26 (74.3)	0.1677
Peripheral artery disease	23 (11.0)	17 (14.0)	4 (7.5)	2 (5.7)	0.1025
Chronic kidney disease	33 (15.8)	14 (11.5)	12 (22.6)	7 (20.0)	0.3949
Arterial hypertension	168 (80.3)	103 (85.1)	39 (73.5)	26 (74.3)	0.0213
Diabetes mellitus	57 (27.2)	36 (29.7)	10 (18.8)	11 (31.4)	0.3468
Autoimmune disease	31 (14.8)	15 (12.3)	8 (15.0)	8 (22.9)	0.6453

The most common comorbidity in all data sets was coronary artery disease (81.8% of total) and arterial hypertension (80.3% of total). Although there were significant differences in preexisting lung disease (e.g., chronic obstructive lung disease, emphysema), all groups had a fairly low number. In summary, the groups were comparable in their baseline demographic data.

### ICU treatment variables

Next, we investigated whether the different dosing strategies differed in their disease severity. Interestingly, 28-day mortality was significantly lower in the cohort treated with bolus injection first with subsequent continuous infusion (53.1%), compared to the other dosing strategies (bolus only: 71.2%; continuous infusion only: 74.3%; Table 2). We also assessed short-term mortality, which we defined as death within 12 h after administration of methylene blue. The differences in 28-day and short-term mortality were not due to differences in disease severity, as SAPSII, APACHE and SOFA scores were similar between the groups. However, patients with bolus + continuous infusion support underwent longer mechanical ventilator support.

**Table 2:** ICU variables.

	**Total (*n* = 209)**	**Bolus + infusion (*n* = 111)**	**Bolus only (*n* = 59)**	**Continuous infusion only (*n* = 39)**	***p* values**
28-day mortality n (%)	130 (62.2)	59 (53.1)	42 (71.2)	29 (74.3)	0.0154
SAPS II (mean ± SD)	59.5 ± 12.0	59.5 ± 12.5	60.4 ± 12.6	61.9 ± 9.4	0.2091
APACHE II (mean ± SD)	25.8 ± 6.4	24.8 ± 6.1	26.9 ± 7.4	27.0 ± 5.4	0.0529
SOFA (mean ± SD)	11.3 ± 3.0	11.04 ± 3.1	11.3 ± 3.3	12.0 ± 2.5	0.2313
Renal replacement therapy in the ICU *n* (%)	183 (87.5)	98 (88.2)	50 (84.7)	35 (89.7)	0.7212
Median LOS-ICU of survivors (interquartile range)	22 (10–43)	11 (4–25)	7 (1–16)	4 (2–14)	0.0067
Median hours of ventilator support (interquartile range)	114 (37–283)	160 (50–334)	113 (24–279)	66 (24–220)	0.0304^#^
**Cumulative dose of methylene blue**					
Methylen blue (mg/kg)	5.04 ± 3.2	6.7 ± 3.2	2.7 ± 1.9	4.03 ± 2.5	< 0.0001
**Initial doses of vasoactive substances at methylene blue administration**					
VIS (interquartile range) [μg/kg/min]	59.8 (39.2–82.4)	58.0 (37.3–80.7)	60.3 (39.8–91.4)	59.6 (41.8–80.5)	0.4243
Norepinephrine treatment *n* (%)	209 (100)	111 (100)	59 (100)	39 (100)	1.0000
Norepinephrine dose (interquartile range) [μg/kg/min]	0.47 (0.30–0.73)	0.46 (0.28–0.71)	0.53 (0.32–0.77)	0.45 (0.32–0.68)	0.2662
Epinephrine treatment *n* (%)	56 (27)	32 (29)	13 (22)	11 (28)	0.6202
Epinephrine dose (interquartile range) [μg/kg/min]	0.043 (0.02–0.10)	0.037 (0.0–0.07)	0.088 (0.02–0.11)	0.10 (0.033–0.129)	0.8809
Vasopressin treatment *n* (%)	191 (91)	105 (94)	49 (83)	37 (94)	0.0265
Vasopressin dose (interquartile range) [U/kg/min]	3 (2–4)	3 (2–4)	3 (2–3)	3 (2–4)	0.5437
Dobutamine treatment *n* (%)	40 (19)	21 (18)	9 (15)	10 (25)	0.4395
Dobutamine dose (interquartile range) [μg/kg/min]	5.0 (3.1–6.21)	4.9 (2.8–6.0)	5.59 (2.9–8.2)	5.0 (4.0–6.2)	0.9533
Milrinon treatment n (%)	118 (56)	66 (59)	32 (54)	20 (51)	0.6218
Milrinon dose (interquartile range) [μg/kg/min]	0.44 (0.3–0.5)	0.41 (0.3–0.5)	0.42 (0.3–0.5)	0.5 (0.4–0.6)	0.4052
**Point of care diagnostics**					
Lactate (interquartile range) [mmol/L]	5.9 (3.3–11.2)	5.5 (3.1–9.8)	6.1 (4–10.7)	9.1 (3.5–12.9)	0.7743
pH (interquartile range)	7.32 (7.27–7.38)	7.33 (7.27–7.38)	7.31 (7.27–7.37)	7.32 (7.27–7.39)	0.6371

# Compared using Wilcoxon rank sum test.

Next, we analyzed the differences in the support of vasoactive substances. As expected, patients with bolus + continuous infusion received the highest cumulative dose of methylene blue. All patients received more than one vasoactive substance. In our institution, we regularly use norepinephrine, epinephrine, vasopressin, milrinone and dobutamine for the treatment of hemodynamic instability. We found that at the beginning of methylene blue therapy, all patients received a comparable degree of pharmacological hemodynamic support (Table 2). Indicators of decreased perfusion, such as lactate and blood pH, were not significantly different between the groups.

### Methylene blue dosing strategy, hemodynamic parameters, and vasopressor requirements

**Figure 2:**
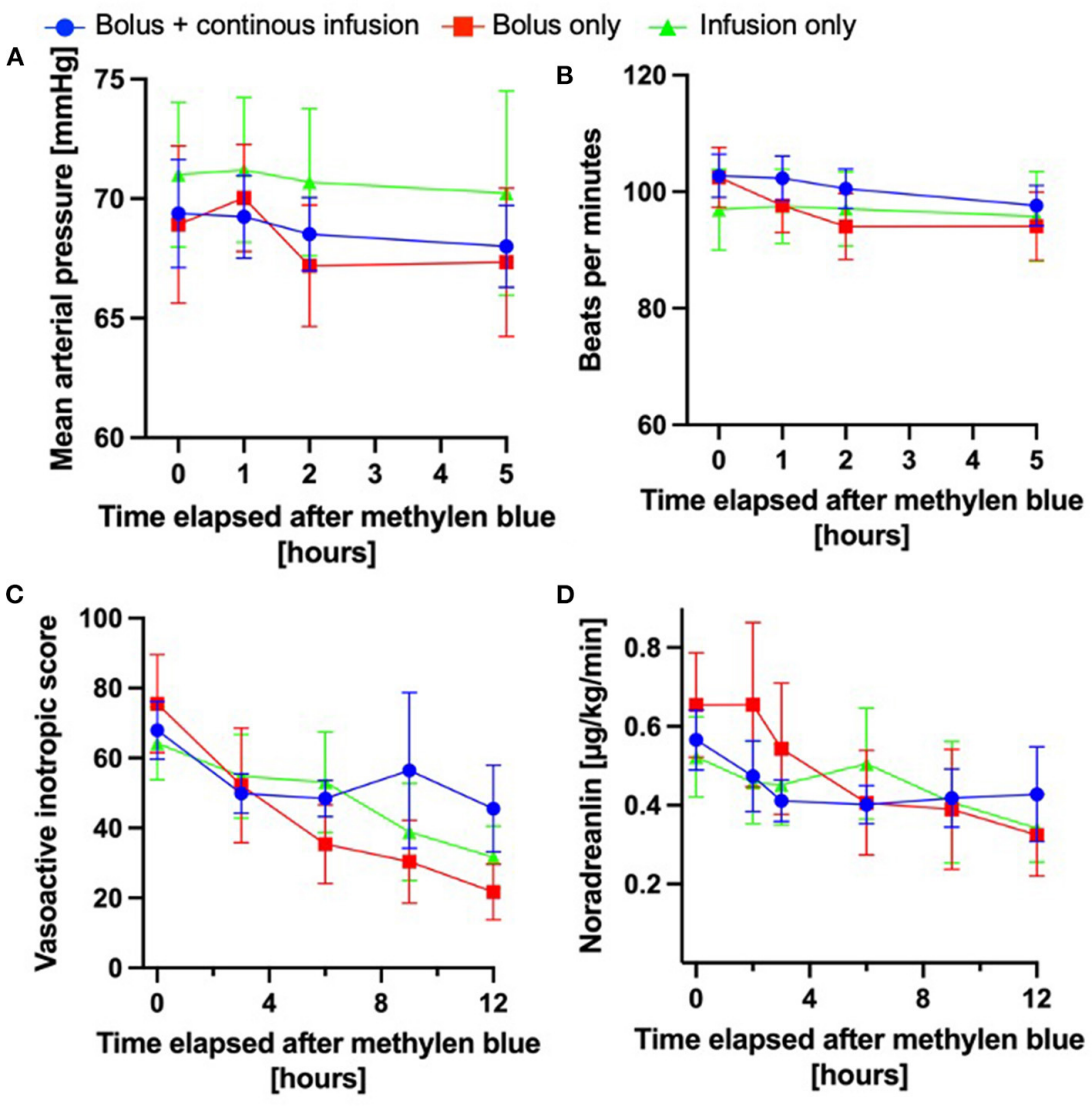
Hemodynamic changes after methylene blue administration. The injection time points of the methylene blue bolus were defined as “0 h”; all subsequent time indicators were defined in relation to the administration of methylene blue administration. **(A)** Mean arterial pressure over time in the different methylene blue treatment groups over time. **(B)** Heart rate over time after methylene blue administration (**A** + **B**: bolus + continuous infusion: *n* = 111–110; bolus only: *n* = 47–59; continuous infusion only *n* = 35–39). **(C)** Norepinephrine infusion rate in μg/kg/min over time after methylene blue administration. **(D)** Development of vasoactive inotropic score after methylene blue administration (**C** + **D**: bolus + continuous infusion: *n* = 111–110; bolus only: *n* = 47–59; continuous infusion only *n* = 35–39); All data are shown as mean ± 95% confidence interval.

To understand whether dosing strategy influences the hemodynamic response to methylene blue administration, we analyzed the basic hemodynamic parameters of mean arterial pressure and heart rate (0, 1, 2, and 5 h), after methylene blue (Figures 2A,B). The hemodynamic response was similar between the three different dosing strategies. In a subset of patients, systemic vascular resistance (SVR) was measured by pulmonary artery catheterization or transpulmonary thermodilution. The evaluation of the SVR measurement closest to the administration of methylene blue and 12 h later did not vary between the different dosing strategies. In a subset of patients (*n* = 157), advanced hemodynamic monitoring (such as pulmonary artery catheter or transpulmonary thermodilution) was used. Among these patients, cardiac index was determined. At the administration of methylene blue, the median cardiac index was 2.97 (interquartile range 2–31 – 3.61), which remained essentially unchanged over 12 h after methylene blue administration (2.86; interquartile range 2.14–3.52). Furthermore, the cardiac indices were not different between the different treatment regimens (0 h: *p* = 0.0714; 12 h *p* = 0.1629).

Moreover, we investigated whether the vasopressor requirements changed differently over time according to the dosing. As shown in Figures 2C,D the doses of norepinephrine (used in 100% of patients) or VIS decreased over time in response to the administration of methylene blue in all groups. We found the largest relative change from VIS 0 h to VIS 3 h (ΔVIS) in the patient cohort treated with a methylene blue bolus only (ΔVIS 29 62%); however, this did not differ significantly from the other cohorts (bolus + continuous infusion ΔVIS 29 ± 33%; continuous infusion only 11 ± 37%; one-way ANOVA *p* = 0.1184).

In summary, all methylene blue treatment regimens are equally effective in promoting hemodynamic stabilization.

### Response rate to methylene blue

**Figure 3:**
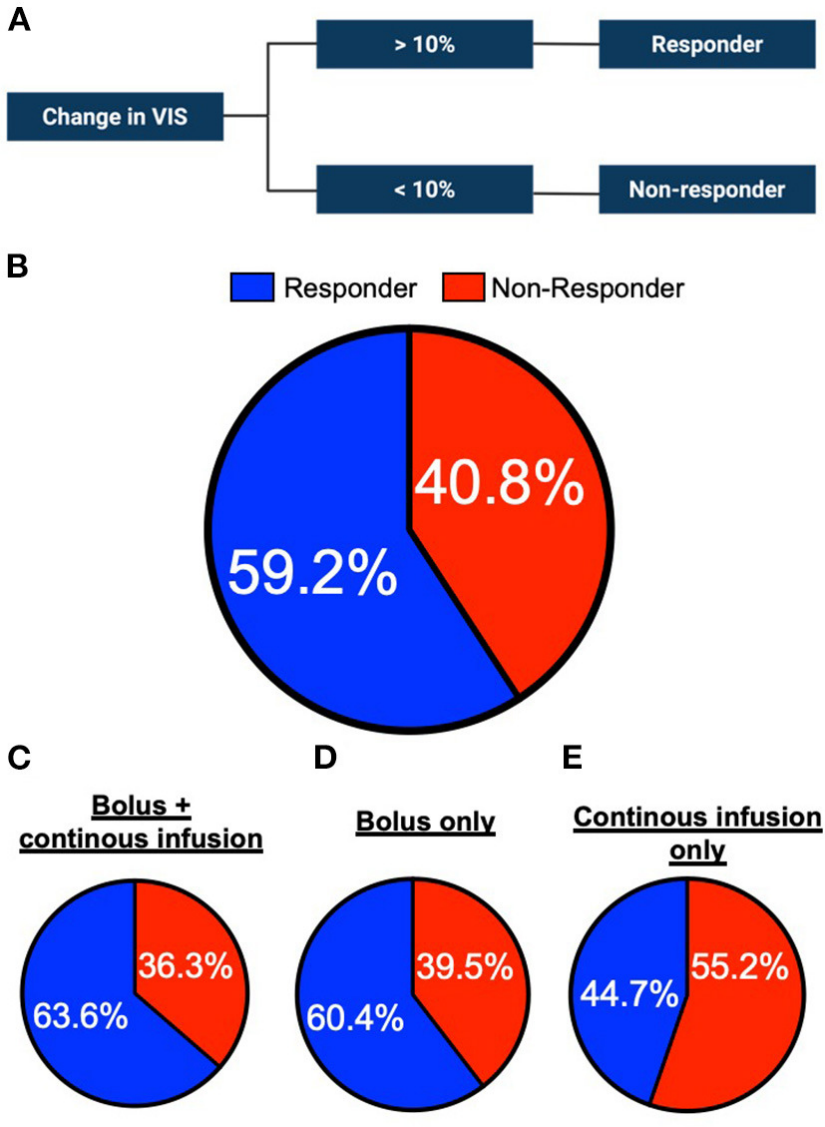
Percentage of methylene blue responders per administration group. **(A)** Schematic of the definition of responder or non-responder status to methylene blue treatment. **(B)** Proportion of responders and non-responders in the study cohort (*n* = 196 in total) **(C)** bolus + continuous infusion cohort (*n* = 110 in total) **(D)** bolus only infusion group (*n* = 48) or **(E)** continuous only cohort (*n* = 38).

Methylene blue improves the mean arterial pressure and decreases the requirement for vasopressors in critically ill patients. However, the response to methylene blue remains unpredictable and a proportion of patients remain unresponsive to administration. We investigated whether the response to methylene blue varies between different treatment strategies. To do this, we analyzed the proportion of clinical responders within the respective group (Figure 3A). In the total cohort, 59.2% of the patients responded to methylene blue, with comparable percentages found in the bolus + continuous infusion and bolus groups (Figures 3B–D). On the contrary, continuous infusion yielded a lower percentage of responders (44.7%, Figure 3E). In a nominal logistic regression analysis, the cumulative dose of methylene blue was not correlated with response status (Odds ratio 1.03, 95% CI 0.94–1.13; *p* = 0.4568).

Next, we investigated whether clinical variables assessed at the bedside are associated with a response to methylene blue. As shown in Table 3, cardiogenic shock and norepinephrine were associated with a higher chance of responding to methylene blue administration, while the bolus treatment regimen was not significantly associated with response.

**Table 3:** Factors associated with methylene blue response in the logistic regression.

	**Univariate logistic regression**		**Multivariate logistic regression**	
	**Odds ratio (95%CI)**	***p*-value**	**Odds ratio (95%CI)**	***p*-value**
**ICU variables on day of methylene blue treatment**				
Age	0.97 (0.22–4.26)	0.9686		
Sex	1.65 (0.84–3.26)	0.1378		
SAPSII	0.99 (0.97–1.01)	0.7118		
Septic shock	0.67 (0.37–1.12)	0.1903		
Cardiogenic shock	**2.10 (1.09–4.05)**	***0.0230***	**2.21 (1.11–4.36)**	***0.0224***
Vasoplegia of other causes	0.77 (0.41–1.41)	0.4010		
**Point-of-care 0h after methylene blue treatment**				
Lactate	1.00 (0.95 to 1.06)	0.7722		
pH	0.38 (0.009 to 15.27)	0.6122		
**Vasopressors/inotropes 0 h after methylene blue treatment**				
Vasoactive Inotropic Score	**1.01 (1.00–1.02)**	***0.029***		
Norepinephrine μg/kg/min)	**3.75 (1.46–9.62)**	***0.0023***	**3.64 (1.39–9.51)**	***0.0083***
Dobutamin μg/kg/min)e	**1.00 (0.95–1.05)**	***0.0317***		
Vasopressin μg/kg/min)	1.21 (0.95–1.54)	0.0938		
Milrinon μg/kg/min)	0.17 (1.28–5.77)	0.0794		
**Hemodynamic variables**				
Mean arterial pressure	1.00 (0.97–1.02)	0.8052		
Heart rate	0.99 (0.98–1.01)	0.8175		
Heart index	1.22 (0.88–1.74)	0.1939		
Systemic Vascular Resistance	0.99 (0.99–1.00)	0.2189		
**Dosing strategy**				
Bolus + continuous infusion	1.52 (0.85–2.7)	0.1517		
Bolus only	**0.48 (0.23–0.98)**	***0.0452***	**0.46 (0.22–0.99)**	***0.0483***
Continuous infusion only	1.07 (0.55–2.08)	0.8413		

Italic letters indicate statistical significance.

In the multivariate model, cardiogenic shock, norepinephrine dose at the time of methylene blue administration, and treatment with bolus alone (negative association) remained independently associated with response to methylene blue (Table 3). ROC analysis displayed a discrimination with an AUC of 0.68551. The goodness-of-fit was appropriate (Hosmer-Lemeshow; *p* = 0.8851).

### Variables associated with mortality at 28 days

The association of variables with mortality at 28 days in the logistic regression analysis is shown in Table 4. In the univariate analysis, we found that SAPSII, lactate at the start of methylene blue administration, blood pH, and higher VIS (translated as higher doses of norepinephrine and vasopressin doses) were associated with mortality at 28 days. Interestingly, the administration of methylene blue as bolus + continuous infusion was associated with decreased mortality at 28 days. In a multivariate model, SAPSII, lactate, and methylene blue dosing strategies remained independently associated with mortality 28 days after methylene blue administration (AUC 0.8054). The Hosmer-Lemeshow test showed appropriate goodness-of-fit (*p* = 0.8180).

**Table 4:** Factors associated with mortality at 28 days in logistic regression.

	**Univariate logistic regression**		**Multivariate logistic regression**	
	**Odds ratio (95%CI)**	***p*-value**	**Odds ratio (95%CI)**	***p*-value**
**ICU variables on day of methylene blue treatment**				
Age	2.91 (0.69–12.3)	0.1450		
Sex	1.45 (0.75–2.80)	0.2622		
**SAPSII**	**1.07 (1.04**–**1.10)**	*** < 0.001***	**1.08 (1.04**–**1.12)**	*** < 0.0001***
Septic shock	1.54 (0.86–2.76)	0.1424		
Cardiogenic shock	1.12 (0.61–2.06)	0.7118		
Vasoplegia of other causes	0.55 (0.30–1.01)	0.0561		
**Point-of-care 0h after methylene blue treatment**				
Lactate	**1.18 (1.09**–**1.26)**	*** < 0.001***	**1.16 (1.08**–**1.25)**	*** < 0.0001***
pH	**0.0014 (3,123E-5**–**0.071**	**0.0006**	0.08 (0.0006–10.05)	0.3054
**Vasopressors/inotropes 0 h after methylene blue treatment**				
Vasoactive Inotropic Score	**1.01 (1.00**–**1.02)**	**0.0004**		
Norepineprhine (μg/kg/min)	**4.56 (1.75**–**11.88)**	***0.0004***	3.06 (0.97–9.64)	0.0559
Dobutamine (μg/kg/min)	1.03 (0.88–1.20)	0.6778		
Vasopressin (μg/kg/min)	**1.28 (1.00**–**1.63)**	**0.0314**		
Milrinon (μg/kg/min)	1.12 (0.15–8.07)	0.9102		
**Hemodynamic variables 0 h after methylene blue treatment**				
Mean arterial pressure	0.99 (0.97–1.02)	0.9077		
Heart rate	1.00 (0.98–1.01)	0.6100		
Heart index	0.97 (0.71–1.32)	0.8704		
Systemic vascular resistance	1.00 (0.99–1.00)	0.6818		
**Dosing strategy of methylene blue**				
Bolus + continuous infusion	**0.43 (0.24**–**0.76)**	***0.0039***	**0.45 (0.23**–**0.90)**	***0.0246***
Bolus only	1.98 (0.90–4.32)	0.0759		
Continuous infusion only	1.74 (0.90–3.33)	0.8889		

Italic letters indicate statistical significance.

In summary, regarding the methylene blue treatment regimen, only bolus administration with continuous infusion was associated with a reduced mortality at 28 days.

## Discussion

In this retrospective cohort study, we found that methylene blue reduces the requirement for vasoactive substances, regardless of the dosing strategy. When analyzing the factors associated with the response to methylene blue, we found that the response rate to methylene blue increased in patients with cardiogenic shock and with higher doses of norepinephrine. Administration of a methylene blue bolus without subsequent continuous infusion reduced the response to methylene blue. The administration of methylene blue as a bolus followed by continuous infusion decreased 28-day mortality. Importantly, no serious undesired effects were observed in our cohort. We only noticed minor undesired drug effects such as skin or urine discoloration.

Shock remains a significant problem in critically ill patients, with high inherent mortality. An international consensus conference defined shock as a “*life-threatening generalized maldistribution of blood flow that results in the inability to deliver and/or use an adequate amount of oxygen, leading to tissue dysoxia”* (16). Numerous basic science and clinical studies investigated the underlying dysregulation of endogenous pathways that sustain defective hemodynamics in shock. Nitric oxide has gained substantial research interest because it influences multiple pathways associated with the development of shock. In homeostasis, NO regulates blood flow in resistance vessels (17), influences coagulation, and modifies inflammatory pathways. During shock, NO production increases dramatically up to 1000 times (1, 18).

In 1992, a case series reported that hemodynamics improved in patients with shock after patients received the NO pathway inhibitor methylene blue (19). Since then, several prospective randomized controlled trials have investigated whether methylene blue reduces vasopressor requirements (11, 12, 19, 20). One trial reported that in patients with vasoplegia after cardiac surgery, mortality in the methylene blue treated group decreased to 0 vs. 21.4% in the placebo treated group (12). However, the study included only 28 patients per group, limiting the generalizability of the trial.

Based on the mechanism of proposed effect in hemodynamic instability (inhibition of NO-production), it is surprising that success of methylene blue treatment was not superior in vasoplegic shock. This suggests that methylene could elicit beneficial effect different from NO-inhibition. One potential explanation could be that methylene blue acts as an antioxidant, similar to vitamin C. Shock patients suffer from an overall depletion of antioxidants (21), and administration of antioxidants have been suggested as a therapeutic strategy (22). In fact, antioxidants reduce the oxidative stress parameter, and plasma levels of the pro-inflammatory cytokine IL-6 in an animal with septic shock (23).

In our cohort, the ICU scoring systems on the day of methylene blue treatment implied a mortality of 50–70% in all groups based on the mean of SAPSII, APACHE III and SOFA. Interestingly, the measured mortality at 28 days in patients receiving a bolus + continuous infusion was at the lower end of the predicted mortality compared to the other dosing regimens. In a univariate and multivariate logistic regression analysis, bolus administration followed by continuous infusion was associated with a lower mortality at 28 days. Interestingly, the cumulative dose of methylene blue, did not differ between the different treatment regimen groups. This could suggest that not the amount of methylene blue *per se*, but also pharmacokinetic properties could influence the response to methylene blue. As such, this observation is well known in critically ill patients in an entirely different context, e.g., treatment with beta-lactam anti-infective drugs. Here, the clinical response rate also increases when substances are administered with a continuous infusion. The cumulative dose does not differ between the different forms of administration. Therefore, the observed effect that methylene blue response increases with a bolus injection (corresponding to a loading dose in anti-infective therapy) and subsequent continuous infusion, could point into the direction of a pharmacodynamic effect that influence the effect of methylene blue. However, prospective studies assessing this phenomenon are lacking.

Interestingly, the reduction in mortality in our cohort appeared to be not related to vasopressor requirements because the three groups experienced similar hemodynamic stabilization. Methylene blue as a bolus and subsequent continuous infusion could cause a sustained inhibition of the NO pathway, as methylene blue blocks the NO pathway in two stages: it inhibits both NO synthase (24) and the downstream effector enzyme, soluble guanylate cyclase (sGC) (25). However, methylene blue also interferes with numerous other pathways (26), and therefore the beneficial properties observed could induce effects in addition to hemodynamics or NO-path inhibition alone. In fact, inhibition of NO-synthase leaves shock mortality unchanged (27). The methylene blue concentration in whole blood exceeds the plasma concentration, indicating that the substance accumulates in blood cells. In acute inflammation, immune cells, such as macrophages, release NO (25), thus enhancing the inflammatory response (28). The accumulation of methylene blue in these cells could dampen an overshooting inflammatory response. In particular, in septic shock, NO overproduction contributes to hypotension, cardiodepression, and vascular hyporeactivity (29). Therefore, the observed effects of methylene blue could modulate inflammatory responses and subsequently reduce mortality.

In our analysis, the response to methylene blue administration was correlated with the diagnosis of cardiogenic shock. This seems counterintuitive at first because, due to the mechanism of the proposed action, most of the studies investigated methylene blue in vasoplegia. However, there is some evidence that increased NO release enhances the detrimental spiral of events in cardiogenic shock. As such, NO blocks the adrenergic response of the myocardium and reduces myocardial contractility (30). Similarly, NO concentration increases in the peripheral blood of patients with acute myocardial ischemia (31). Furthermore, 20% of patients with underlying cardiogenic shock experience a systemic inflammation response syndrome, and the majority show a positive blood culture with low systemic vascular resistance (32). This provides an explanation why patients with the primary diagnosis of cardiogenic shock could benefit from methylene blue treatment.

## Limitations

Finally, our study has several important limitations. Our results should be seen in the context of the study design. First, we performed our study under the assumption that methylene blue decreases the vasopressor requirements. We have not included a control group with shock and without methylene blue treatment, thus our study cannot answer cannot provide evidence if methylene reduces mortality *per se*. However, our findings suggest that within differences could exist between different administration protocols. Being retrospective in design, our study has the inherent limitation that we cannot account for all variables that impacted the prescription and timing of methylene blue administration. As such, blood transfusion or volume resuscitation could have changed the findings of our study. Nevertheless, disease severity and forms of shock were similar between all groups, which suggests that differences between different treatment groups were comparable. In addition, we did not assess the length of stay in the ICU prior to methylene blue treatment. Another important limitation is the size of the individual cohorts. Even though we did not detect a significant difference in demographic parameters or in ICU scoring systems, the numeric differences between the groups are notable. Moreover, we did not perform retrospective matching (such as propensity score matching) as this technique does not account for unmeasured characteristics and cofounders (33).

Despite these limitations, our study adds to the body of evidence on the effect of methylene blue on shock. To our knowledge, our study presents the largest cohort of methylene blue treatment in ICU patients to date. Thus, we have concluded that methylene blue is safe in this population and increases survival in some patients.

## Conclusion

In summary, we report the results of the largest cohort of methylene blue-treated ICU patients to date. In our single-center, retrospective analysis, methylene blue administration was associated with a reduced 28-day mortality in critically ill patients with shock, when administered as a bolus followed by a continuous infusion. However, prospective randomized studies are needed to systematically evaluated the values of methylene blue in the treatment of shock.

## Data availability statement

The original contributions presented in the study are included in the article/supplementary material, further inquiries can be directed to the corresponding author.

## Ethics statement

The approval for this study was obtained from the local institutional review board and the Ethics Committee of the Tübingen University Hospital Tübingen (549/2019BO2).

## Author contributions

SS-Y, PW, and MKo designed the study. MKo performed data analysis and interpretation, and statistical data analysis. HM and MKe performed statistical analysis and helped to write the manuscript. Y-SF cross-checked statistical analysis. K-LH-S, CS, and PR contributed to the manuscript preparation, drafting, critique, and review. All authors approved the final version of the manuscript submitted.

## Conflict of interest

The authors declare that the research was conducted in the absence of any commercial or financial relationships that could be construed as a potential conflict of interest.

## Publisher's note

All claims expressed in this article are solely those of the authors and do not necessarily represent those of their affiliated organizations, or those of the publisher, the editors and the reviewers. Any product that may be evaluated in this article, or claim that may be made by its manufacturer, is not guaranteed or endorsed by the publisher.
