# Corrigendum: Methylene blue dosing strategies in critically ill adults with shock—A retrospective cohort study

**DOI:** 10.3389/fmed.2022.1094735

**Published:** 2022-11-23

**Authors:** Sibel Sari-Yavuz, Ka-Lin Heck-Swain, Marius Keller, Harry Magunia, You-Shan Feng, Helene A. Haeberle, Petra Wied, Christian Schlensak, Peter Rosenberger, Michael Koeppen

**Affiliations:** ^1^Department of Anesthesiology and Intensive Care Medicine, University Hospital Tübingen, Tübingen, Germany; ^2^Institute for Clinical Epidemiology and Applied Biostatistics (IKEaB), Eberhard-Karls-University Tübingen, Tübingen, Germany; ^3^Department of Thoracic and Cardiovascular Surgery, University Hospital Tübingen, Tübingen, Germany

**Keywords:** shock, methylene blue, critical care, retrospective study, clinical trial

In the published article, there was an error. The dosing of the continuous infusion of methylene blue is incorrect. Currently the text states “continuous infusion with 0.25 mg/kg/min” this should be changed to “continuous infusion with 0.25 mg/kg/h”.

A correction has been made to **Methods**, *Methylene blue therapy*, second paragraph. This sentence previously stated:

“Three different dosing strategies have been used in our institution in the past: bolus injection alone, continuous infusion without bolus, injection, or bolus injection followed by continuous infusion (bolus 2 mg/kg; continuous infusion with 0.25 mg/kg/min).”

The corrected sentence appears below:

“Three different dosing strategies have been used in our institution in the past: bolus injection alone, continuous infusion without bolus, injection, or bolus injection followed by continuous infusion (bolus 2 mg/kg; continuous infusion with 0.25 mg/kg/h).”

The authors apologize for this error and state that this does not change the scientific conclusions of the article in any way. The original article has been updated.

## Publisher's note

All claims expressed in this article are solely those of the authors and do not necessarily represent those of their affiliated organizations, or those of the publisher, the editors and the reviewers. Any product that may be evaluated in this article, or claim that may be made by its manufacturer, is not guaranteed or endorsed by the publisher.

